# Decreased transthyretin predicts a poor prognosis in primary myelodysplastic syndrome

**DOI:** 10.3389/fnut.2023.1125768

**Published:** 2023-03-07

**Authors:** Ying Chen, Tingting Niu, Ting Chen, Yue Wu, Duobing Zou, Cong Shi, Ying Wu, Zhaoyi Zhang, Ningning Wu, Yi Zhang, Xiao Yan, Lixia Sheng, Dingfeng Lv, Guifang Ouyang, Xueqin Chen, Qitian Mu

**Affiliations:** ^1^Laboratory of Stem Cell Transplantation, Ningbo First Hospital, Ningbo, Zhejiang, China; ^2^Ningbo Clinical Research Center For Hematologic Malignancies, Ningbo, Zhejiang, China; ^3^Hematology Department, Ningbo First Hospital, Ningbo, Zhejiang, China; ^4^Medical School of Ningbo University, Ningbo, China; ^5^Department of Blood Transfusion, Ningbo First Hospital, Ningbo, China; ^6^Department of Traditional Medicine, Ningbo First Hospital, Ningbo, Zhejiang, China

**Keywords:** myelodysplastic syndromes, transthyretin, prognosis, IPSS-R, IPSS-M

## Abstract

**Background:**

This study aims to investigate the prognostic significance of transthyretin in newly diagnosed myelodysplastic syndromes (MDS).

**Methods:**

The clinical, laboratory, and follow-up data of 280 newly diagnosed patients with MDS were collected. The relationship between serum transthyretin levels and overall survival (OS) and leukemia-free survival (LFS) were analyzed by Kaplan–Meier analysis and Cox Regression Model.

**Result:**

In the MDS cohort, there were 121 cases in the low transthyretin group and 159 cases in the normal transthyretin group. MDS patients with decreased transthyretin had a higher risk score on the Revised International Prognostic Scoring System (IPSS-R) (*p* = 0.004) and on the molecular IPSS (IPSS-M) (*p* = 0.005), a higher frequency of TP53 mutation (*p* < 0.0001), a shorter OS (*p* < 0.0001) and LFS (p < 0.0001). Multivariate analyses showed that higher IPSS-R and IPSS-M score were adverse factors for OS (*p* = 0.008 and *p* = 0.015, respectively) and LFS (*p* = 0.024 and *p* = 0.005, respectively). Mutations of TP53 and NRAS were also poor factors for LFS (*p* = 0.034 and *p* = 0.018, respectively). Notably, decreased transthyretin was an independent adverse predictor for OS (*p* = 0.009, HR = 0.097, 95%CI, 0.017–0.561) but not for LFS (*p* = 0.167) when IPSS-R was included in the Cox regression model and an independent poor one for OS (*p* = 0.033, HR = 0.267, 95%CI, 0.080–0.898) and LFS (*p* = 0.024, HR = 0.290, 95%CI, 0.099–0.848) while IPSS-M involved.

**Conclusion:**

The results indicate that decreased transthyretin could be an independent adverse prognostic factor in patients with MDS and may provide a supplement to IPSS-R and IPSS-M.

## 1. Introduction

Myelodysplastic syndromes (MDS) are a heterogeneous group of clonal diseases originating from hematopoietic stem cells. These diseases are characterized by dysplasia in one or more cell lines, differentiation disorder, peripheral blood cell reduction, and predisposition to acute myeloid leukemia (AML) progression (1). Several scoring systems, such as the Revised International Prognostic Scoring System (IPSS-R) and the World Health Organization Classification-Based Prognostic Scoring System (WPSS), have been applied to stratify MDS in recent years (2–4). However, monosomal karyotype and some mutations, such as TP53, SF3B1, are independent of these scoring systems and have been reported to measure the prognosis of MDS (5–7). Some novel biomarkers such as serum apolipoprotein A1, caspase-1 and PD-L1 co-expression patterns to predict MDS risk have also been discovered (8, 9). Recently, a new prognostic scoring system, molecular IPSS (IPSS-M), was introduced by international working group for the prognosis of MDS (IWG-PM) (10). But molecular testing is not yet routine globally because of limit of cost or laboratory conditions. Therefore, it is still necessary to explore new and simple prognostic factors to evaluate the prognostic stratification of MDS.

Transthyretin (transthyretin), also called pre-albumin, is synthesized by the liver. It can binds thyroxine so it is better known as thyroxine-binding prealbumin (TBPA). Also, transthyretin combines with retinol-binding protein 4 (RBP4) and plays an important role in transporting vitamin A (11). The half-life of transthyretin is about 2 days, which is much shorter than that of albumin (21 days). Thus, transthyretin is considered as a more sensitive marker for assessing nutritional status in patients (12).

Previous studies have reported that transthyretin was an independent prognostic factor for urothelial carcinoma, gastric cancer, and non-small cell lung cancer (13–16). A low serum level of albumin has also been reported as an independent prognostic factor for overall survival (OS) and leukemia-free survival (LFS) in MDS (17, 18). However, the prognostic value of transthyretin levels for MDS has not yet been reported. In this study, we retrospectively analyzed the data of 280 MDS patients and explored the potential prognostic value of transthyretin levels.

## 2. Materials and methods

### 2.1. Patients

The data of 280 newly diagnosed patients with MDS at Ningbo First Hospital from June 2009 to July 2021 were collected. Diagnosis and classification of MDS followed the 2016 World Health Organization (WHO) classification (2), and the risk stratifications of MDS followed the IPSS-R and IPSS-M (10, 19).

Among the 280 MDS patients, 58 were treated with demethylation, 43 with chemotherapy, and 169 with symptomatic and supportive treatment. In the cohort, 31 patients underwent hematopoietic stem cell transplantation (HSCT). This study was approved by the Human Ethics Committee of Ningbo First Hospital and complied with the Declaration of Helsinki.

### 2.2. Transthyretin assay

Serum transthyretin levels were assessed by using a turbidimetric immunoassay. The reagents were tested using an automatic biochemical analyzer (Beckman AU5800) according to the instructions of the Kit For transthyretin Assay (Saike, Zhejiang, China).

### 2.3. Cytogenetic analysis

Cytogenetic analysis was performed using R-banding techniques on bone marrow (BM) cells at diagnosis. The results were described according to the International System for Human Cytogenetic Nomenclature (2016) (ISCN2016).

### 2.4. Fluorescence *in situ* hybridization analysis

To determine the copy-number state of TP53 in patients, fluorescence *in situ* hybridization (FISH) was performed on BM cells from cytogenetic cultures according to product instructions using the P53 gene probe (Guangzhou LBP, China). The Signal and image analysis were evaluated by a fluorescence microscope (Olympus BX51, Japan) and an imaging software (IMSTAR, France). Signals were counted at least 200 metaphase or interphase nuclei and deletion of P53 ratio higher than the 5% threshold was regarded as positive.

### 2.5. Gene mutational analysis

The genomic DNA of BM cells from MDS patients was extracted and amplified by AmpliSeq multiplex PCR, and the sample library was constructed. Next-generation sequencing was carried out on the ion proton platform. The analysis was carried out with reference to the cosmic database to determine the mutation sites of pathogenic genes. The average sequencing depth was 2000×, and a variant allele frequency (VAF) > 2% was considered positive. The detection was carried out at Wuhan Kangshengda medical laboratory, and the 14 genes panel included *NRAS, DNMT3A, SF3B1, IDH1, TET2, EZH2, JAK2, CBL, ETV6, IDH2, TP53, SRSF2, ASXL1,* and *RUNX1*. On the basis of 14 genes panel, the 34 genes panel additionally detected *BCOR, BCORL1, CALR, CEBPA, CSF3R, ETNK1, FLT3, KIT, MPL, NF1, NPM1, PHF6, PIGA, PTPN11, SETBP1, STAG2, TET2, U2AF1, WT1,* and *ZRSR2.* Among these patients, 14 genes panel was carried out in 21 patients while 34 genes panel was carried out in 88 patients.

### 2.6. Analysis of survival and disease progression

The last follow-up time was December 31, 2021, and the median follow-up time was 17 (0.03–127) months. OS was defined as the time from diagnosis to death for any reason, the last follow-up, or HSCT. LFS was defined as the time from diagnosis to progression or death from any cause. The follow-up data were collected using inpatient medical records, outpatient medical records, or telephone.

### 2.7. Statistical analysis

Statistical significance was analyzed using the SPSS (version 22.0) software (IBM Corporation, Armonk, NY, United States). Differences in the distribution of continuous variables between groups were analyzed by Mann–Whitney U and categorical variables by chi-squared test. Survival analyses were carried out using the Kaplan–Meier method. Comparisons were performed using the log-rank test and multivariable analyses were conducted using the Cox proportional hazard regression model. All statistical tests were performed with a 95% confidence interval (CI). A *p*-value of <0.05 was considered statistically significant.

## 3. Results

### 3.1. Patients characteristics

A total of 280 patients with MDS at diagnosis were enrolled in the study. These included 166 males and 114 females, with a median age of 63 years (16–91 years) and a median survival time of 28 (0.03–127) months. The IPSS-R was used to stratify the prognosis of 240 patients (the remaining 40 patients could not be stratified due to incomplete karyotype data) as follows: 13 cases in the very good group, 44 in the good group, 80 in the intermediate group, 52 in the poor group, and 51 in the very poor group. While the IPSS-M was also used to stratify the prognosis of 106 patients, including 8 patients in the very low group, 15 in the low group, 12 in the moderate low group, 20 patients in the moderate high group, 21 patients in the high group, and 30 patients in the very high group. Among them, 34 patients progressed into acute leukemia, 136 died, and 30 underwent transplantation. We defined patients with transthyretin <20 mg/dL as the group with low transthyretin (121 patients). Patients with transthyretin ≥20 mg/dL were defined as the group with normal transthyretin, and 159 patients were enrolled. Our cohort’s median transthyretin value at initial diagnosis was 21.85 mg/dL (range: 4–58 mg/dL).

### 3.2. Clinical and laboratory characteristics in low transthyretin patients

In the cohort, compared with the normal group, the low transthyretin group had older age, higher BM blast count, higher globulin and lower hemoglobin (HB) level, lower platelet (PLT) count, lower albumin level, and lower albumin-to-globulin ratio (AGR). Also, the low transthyretin group had a higher ratio of IPSS-R poor and very poor subgroups (*p* < 0.05). Additionally, the distribution of 2016 WHO subtypes between the two groups tended to have a significant difference (*p* = 0.056). We also analyzed the relationship between the transthyretin level and MDS-SF3B1 and MDS-biTP53, which are subtypes newly proposed in 2022 WHO. Though the proportion of MDS-SF3B1 patients in the low transthyretin was less than the nromal group, there was no significant difference (*p* = 0.16). Notably, all MDS-biTP5 patients were detected in low transthyretin group and there was a significant difference between the two groups (*p* = 0.014). However, there were no significant differences in gender, absolute neutrophil count (ANC), and serum ferritin (SF) levels (Table 1).

**Table 1:** Transthyretin level in relation to clinical and laboratory features in MDS patients.

Characteristic	All patients	Low transthyretin	Normal transthyretin	*p*-value
Gender, n (%)				
Male	166(59.3)	70(57.9)	96(60.4)	0.713
Female	114(40.7)	51(42.1)	63(39.6)	
Age, years median (range)	63(16–91)	68(19–91)	61(16–89)	<0.001
ANC, (×109/L) median (range)	1.2(0–7.4)	1.1(0.1–7.4)	1.2(0–6.9)	0.629
HB (g/L) median (range)	7.45(2.2–14.2)	6.8(2.3–13.6)	8.4(2.2–14.2)	<0.0001
PLT (×109/L) median (range)	52(2–434)	46(2–332)	59(6–434)	0.017
Bone marrow blast (%) median (range)	5(0–19.5)	6.5(0–19.5)	4(0–19.5)	0.003
SF, μg/L median (range)	312.7(5.52–2616)	339.4(12.5–2616)	305(5.52–1793)	0.457
Albumin	39.4(18.3–52.7)	35.2(22.4–44.9)	41.5(18.3–52.7)	<0.0001
Globulin	27.6(15.7–56.8)	28.5(15.7–44.7)	26.9(16.5–56.8)	0.027
AGR	1.4(0.6–2.6)	1.2(0.6–2.6)	1.5(0.7–2.6)	<0.0001
IPSS-R risk, n (%)				0.004
Very good	13(5.42)	3(3.12)	10(6.94)	
Good	44(18.33)	12(12.5)	32(22.22)	
Intermediate	80(33.33)	27(28.13)	53(36.81)	
Poor	52(21.67)	23(23.96)	29(20.14)	
Very poor	51(21.25)	31(32.29)	20(13.89)	
IPSS-M risk, n (%)				0.005
Very low	8(7.5)	1(2.9)	7(9.9)	
Low	15(14.2)	1(2.9)	14(19.7)	
Moderate low	12(11.3)	2(5.7)	10(14.1)	
Moderate high	20(18.9)	6(17.1)	14(19.7)	
High	21(19.8)	8(22.9)	13(18.3)	
Very high	30(28.3)	17(48.6)	13(18.3)	
2016 WHO classification, n (%)				0.056
MDS-SLD	20(7.14)	11(9.10)	9(5.66)	
MDS-MLD	80(28.57)	28(23.14)	52(32.7)	
MDS-RS-SLD	10(3.57)	2(1.65)	8(5.03)	
MDS-RS-MLD	8(2.86)	3(2.48)	5(3.15)	
MDS-5q-	7(2.5)	2(1.65)	5(3.15)	
MDS-EB1	60(21.43)	24(19.83)	36(22.64)	
MDS-EB2	80(28.57)	46(38.02)	34(21.38)	
MDS-U	15(5.36)	5(4.13)	9(5.66)	
MDS-SF3B1^&^, n (%)	13(4.6)	3(2.9)	10(5.7)	0.16
MDS-biTP53^&^, n (%)	5(1.8)	32(4.8)	0/0	0.014
Gene mutation*, n (%)	85(78)	32(88.9)	53(72.6)	0.084

*at least one mutation.

& new subtypes proposed in 2022 WHO.

### 3.3. Relationship between gene mutations and decreased transthyretin

The mutation data of 109 MDS patients were available at diagnosis, including 36 patients in the low transthyretin group and 73 patients in the normal transthyretin group.

**Figure 1:**
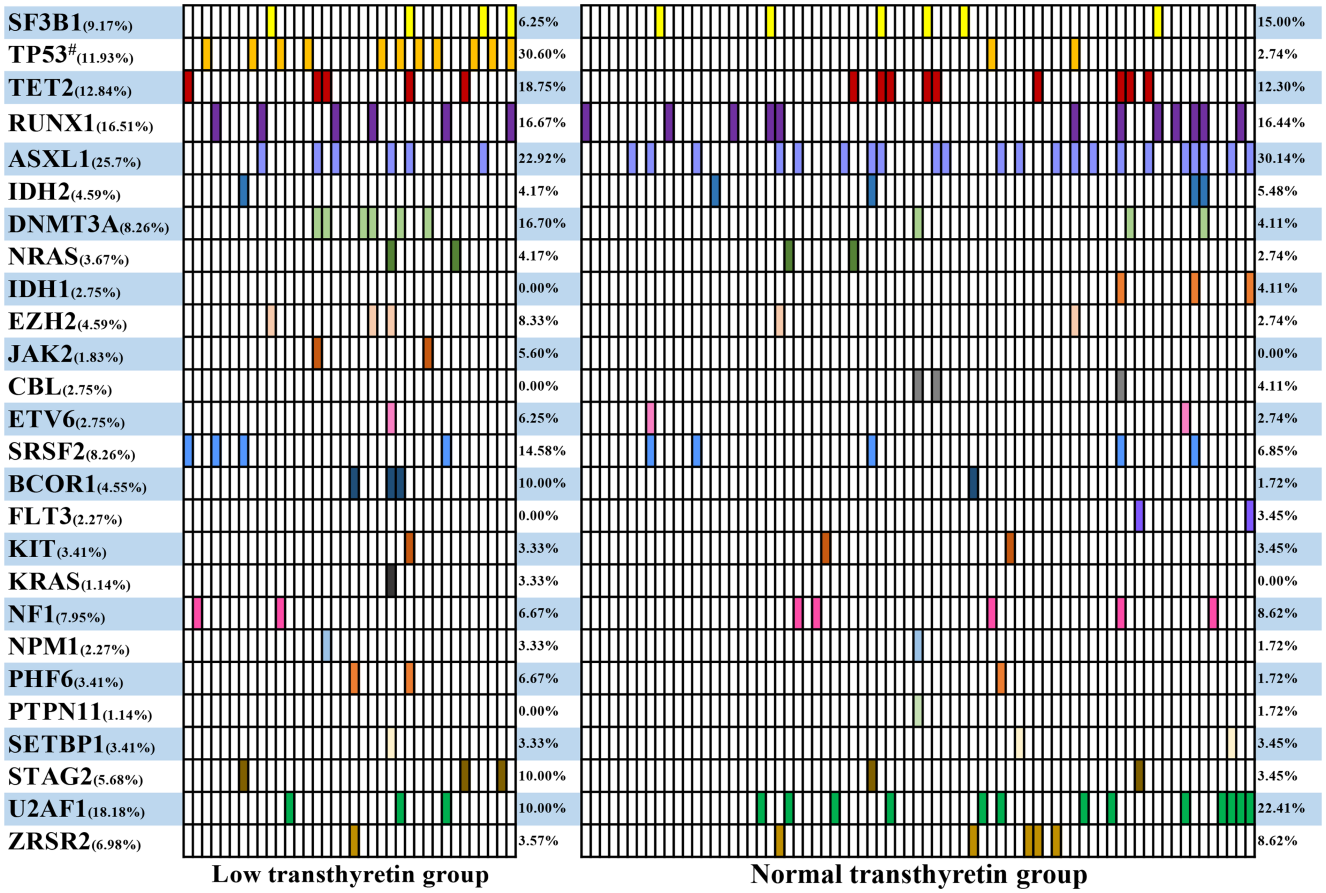
Common genes carrying mutations in MDS patients.

Among these patients, at least one mutation was detected in 85 patients (78.0%), and no mutations were detected in 24 patients (22.0%). The median number of gene mutations was 1 (range 0–7). The distribution of mutations is shown in Figure 1. *ASXL1* mutation appeared the most (25.7%), followed by *U2AF1* mutation (18.18%), *RUNX1* and *TP53* mutations (16.51% and 11.93%, respectively), *TET2* mutation (12.84%), *SF3B1* mutation (9.17%), *SRSF2* and *DNMT3A* mutations (both 8.26%), and *NF1* mutation (7.95%) (Figure 1). The low transthyretin group had a higher ratio of mutations compared to the normal transthyretin group but there was no significant difference between the two groups (88.9% vs. 72.6%, *p* = 0.084). Among these mutations, the low transthyretin group showed a higher mutation frequency of TP53 (30.6% vs. 2.7%, *p* < 0.0001) than the normal transthyretin group (Figure 1). However, we did not detected *BCOR, CALR, CEBPA, CSF3R, ETNK1, MPL, PIGA, WT1* mutations in our cohort.

### 3.4. Decreased transthyretin was associated with shorter overall survival and leukemia-free survival

**Figure 2:**
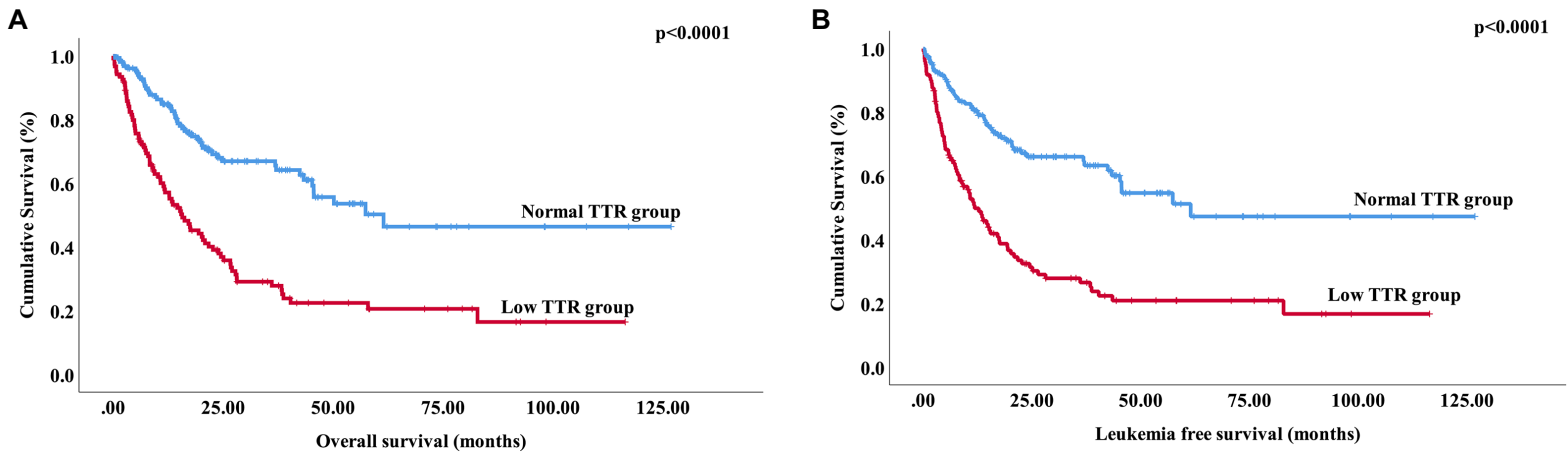
Overall survival and leukemia-free survival of MDS patients stratified according to transthyretin level **(A)** Overall survival of 280 patients with primary MDS stratified by transthyretin <20 mg/dL vs. transthyretin ≥20 mg/dL (*p* < 0.0001). **(B)** Leukemia-free survival of 280 patients with primary MDS stratified by transthyretin <20 mg/dL vs. transthyretin ≥20 mg/dL (*p* < 0.0001).

Among the 280 newly diagnosed patients with MDS, 17 patients in the low transthyretin group progressed to acute leukemia, and 83 patients died. In the normal transthyretin group, 17 patients progressed to acute leukemia, and 53 patients died. The median OS in the low transthyretin group was 15.7 months (range: 0.03–116.4 months), which was significantly shorter than that in the normal transthyretin group (61.47 months, range: 0.3–126.83 months, *p* < 0.0001) (Figure 2A). The median LFS in the low transthyretin group was 11.8 months (range: 0.03–116.4 months), which was also shorter than that in the normal transthyretin group (61.47 months, range: 0.3–126.83 months, *p* < 0.0001) (Figure 2B). Additionally, older age (>60 years), male, higher-risk IPSS-R, higher-risk IPSS-M chemotherapy therapy, lower albumin (<40 mg/dL), lower AGR (<1.2), higher SF (>338 μg/L), TP53 mutation, DNMT3A mutation, and NRAS mutation were associated with shorter OS and LFS. Moreover, MDS patients with EZH2 mutation had a shorter LFS (median LFS: 15.43 vs. 82.83 months, *p* = 0.031).

### 3.5. Multivariate analysis of decreased transthyretin for clinical outcome in MDS

The variables with *p* < 0.1 were included in the multivariate analysis, the result showed that low transthyretin was an independent predictor for poor OS (*p* = 0.009, HR = 0.097, 95%CI, 0.017–0.561) but not for LFS (*p* = 0.167). Additionally, high IPSS-R was an adverse factor for OS and LFS (*p* = 0.008 and *p* = 0.024, respectively) in the multivariate models. High SF levels were associated with shorter OS (*p* = 0.001), and mutations of TP53 and NRAS were factors indicative of poor LFS (*p* = 0.034 and *p* = 0.018, respectively) (Table 2).

**Table 2:** Multivariate analyses involved in IPSS-R for overall survival and leukemia-free survival in 280 patients with primary MDS.

	Univariate analysis for OS *p*-value	Multivariate analysis for OS *p*-value	Univariate analysis for LFS *p*-value	Multivariate analysis for LFS *p*-value
Transthyretin (<20 mg/dL)	<0.0001	0.009	<0.0001	0.167
Age (>60 years)	<0.0001	0.53	<0.0001	0.732
Gender (male)	0.003	0.225	0.006	0.27
IPSS-R risk	<0.0001	0.008	<0.0001	0.024
Treatment methods^※^	0.042	0.623	0.001	0.972
Albumin (<40 mg/dL)	0.001	0.59	<0.001	0.602
Globulin (>40 mg/dL)	0.766	–	0.462	–
AGR (>1.2)	<0.0001	0.454	<0.0001	0.322
SF (>338 μg/L)	0.001	0.001	<0.001	0.092
P53 mutation	<0.0001	0.077	<0.001	0.034
biP53	<0.001		0.002	
DNMT3A mutation	0.008	0.217	<0.001	0.48
NRAS mutation	0.015	0.996	<0.001	0.018
EZH2 mutation	0.13	0.944	0.031	0.255

※ including demethylation, chemotherapy, and supportive treatment.

Next, we incorporated transthyretin (<20 mg/dL), gender (male), IPSS-M risk, treatment methods, albumin (<40 mg/dL), AGR (>1.2), and SF (>338 μg/L) into the IPSS-M multivariate analysis. Transthyretin (<20 mg/dL) and IPSS-M risk retained independent adverse prognostic values for both OS (*p* = 0.033, HR = 0.267, 95%CI, 0.080–0.898) and LFS (*p* = 0.024, HR = 0.290, 95%CI, 0.099–0.848) (Table 3).

**Table 3:** Multivariate analyses involved in IPSS-M for overall survival and leukemia-free survival in 280 patients with primary MDS.

	Univariate analysis for OS *p*-value	Multivariate analysis for OS *p*-value	Univariate analysis for LFS *p*-value	Multivariate analysis for LFS *p*-value
Transthyretin (<20 mg/dL)	<0.0001	0.033	<0.0001	0.024
Gender (male)	0.003	0.329	0.006	0.139
IPSS-M risk	<0.0001	0.015	<0.0001	0.005
Treatment methods^※^	0.042	0.945	0.001	0.509
Albumin (<40 mg/dL)	0.001	0.058	<0.001	0.109
AGR (>1.2)	<0.0001	0.250	<0.0001	0.974
SF (>338 μg/L)	0.001	0.240	<0.001	0.563

※ including demethylation, chemotherapy, and supportive treatment.

## 4. Discussion

This study retrospectively analyzed the data of 280 newly diagnosed MDS patients from 2009 to 2021 to explore the impact of transthyretin levels on the prognosis of patients. Our research suggested that older age, lower HB level, lower PLT count, lower albumin level, lower AGR, higher BM blast count, higher globulin, higher IPSS-R, and higher IPSS-M were associated with decreased transthyretin. Wang et al. (20) reported that HB decreased significantly in acute coronary syndrome (ACS) patients with low transthyretin. In a heart failure study, low transthyretin was reported to be associated with older age and lower albumin and HB (21). The possible reason is that disease and physical fitness affect the body’s nutritional status. In our cohort, there was no significant difference in the distribution of the 2016 WHO subtypes but a obviously difference in the MDS-biTP53 subtype which was only distributed in the low transthyretin group. MDS-biTP53 is a new subtype which is firstly appeared in the 2022 WHO. These persons typically have complex cytogenetics, fewer co-mutations, rapid disease progression and therapy resistance (22). In addition, Chen et al. demonstrated that high transthyretin (variants) could be potential disease-associated markers for del (5q) MDS patients treated by lenalidomide (23). Our analysis showed that more normal transthyretin patients were distributed in the MDS-5q-subtype but unfortunately there was no significant difference.

Patients with malignant tumors often suffer from malnutrition. Recently, increasing evidences indicate a close connection between nutrition, inflammation, immunity, and cancer (24). Cancer-related malnutrition, associated with a poor response to therapy and an adverse prognosis, has a high incidence in patients with cancer (25). Marcos et al. (26) showed that malnutrition impaired the immune system, and suppressed immune functions that were basic to host protection. Prieto et al. (27) demonstrated that immunonutrition formulas could modulate inflammatory and immune responses in cancer patients. Transthyretin is a liver-synthesized protein that is often used to evaluate the nutritional status of patients with malignant tumors (28, 29). The synthesis of transthyretin is suppressed in the inflammatory state and is related to immunity as a negative acute-phase protein, such as tumor necrosis factor (TNF) and interleukin (13, 15). Transthyretin can well reflect the malnutrition status of the body, we supposed that decreased transthyretin might cause chemotherapy resistance, decrease autoimmune function, increase the change of serious infection, and finally shorten the survival of the MDS patients. Recently, a study (30) showed that nutritional interventions are able to improve obviously quality of life in cancer but prolong survival to a very limited degree. However, unlike other types of cancer, MDS patients, mainly occurs in the elderly, have a median OS of 3 years to 5 years. Nutritional interventions could be more important for prolonging survival for MDS patients with malnutrition.

Interestingly, our results from IPSS-R multivariate analyses showed that decreased transthyretin was an independent adverse prognostic factor for OS but not for LFS. However, in the IPSS-M multivariate analyses, decreased transthyretin was an independent adverse prognostic factor for both OS and LFS. IPSS-M which mainly adds data on 16 main effect genes and 15 residual genes mutations based on IPSS-R to classify people with MDS into six survival strata is newly developed by Bernard et al. (10). Wherein, TP53^multihit^, FLT3 mutations, and MLL^PTD^ were strong predictors of adverse outcomes. Compared with the IPSS-R, the IPSS-M resulted in marking increase in model discrimination and improving prognostic accuracy across all long-term clinical end points, including OS and LFS. Recently, WU J et al. reported that IPSS-M took an advantage in subjects ≥60 years MDS patients because of a great frequency of mutations correlated with survival in those ≥60 years patients (31). In our cohort, several gene mutations were incorporated in the IPSS-R multivariate analyses, however we did not analyze TP53^multihit^ and MLL^PTD^ in MDS patients due to lack of enough data.

Previous literature showed that hypoalbuminemia is an independent prognostic factor for OS in MDS patients and is associated with a significantly higher non-relapse mortality and reduced OS for inpatients with AML and MDS at 90 days after allogeneic stem cell transplantation (17, 18, 32). However, although the group with hypoalbuminemia had shorter OS and LFS, the multivariate analyses indicated that it was not an independent adverse prognostic factor. One possible reason is that transthyretin is a more sensitive indicator than albumin in reflecting nutrition.

In our study, we observed that patients with *NRAS* mutations harbored more shorter OS in the low transthyretin MDS patients compared to the normal group. Muhammad et al. (33) showed that MDS patients with RAS mutation easily progressed to AML and RAS mutation was an unfavorable indicator of survival in AML. TP53 mutation is generally associated with complex karyotype, lower platelet count, and elevated BM blast percentage in MDS patients (34). Additionally, our study demonstrated that TP53 mutation is related to low transthyretin and patients with TP53 mutation tend to have shorter OS. The progression time of AML in patients with TP53 mutation was also significantly shortened. Jädersten et al. (35) and Bejar et al. (36) reported that TP53 mutations have a strong and independent prognostic significance for patients with MDS, and patients with TP53 mutation can relapse quickly after various forms of treatment. In recent years, the biological and clinical implications of TP53 allelic state which predicted risk of death and leukemic transformation independently of the Revised International Prognostic Scoring System (IPSS-R) have been investigated in MDS and is associated with complex karyotype, few co-occurring mutations, high-risk presentation and poor outcomes (37). In our group, there was only five MDS-biTP53 patients so that cannot been added into the prognosis analysis. Moreover, DNA methyltransferase enzyme DNMT3A, a DNA methylation regulatory gene whose mutation was an adverse prognostic biomarker in both AML and MDS patients, was detected to be associated with low transthyretin in our cohort (38, 39). These mutations could accelerate the deterioration of nutritional status deterioration and contribute to lower transthyretin in this disease.

The IPSS-R is the most commonly used scoring system for MDS prognosis. In our study, high IPSS-R score was associated with shorter OS and LFS, consistent with previous studies (40, 41). Notably, lower transthyretin could predict an increased risk of death independently of both IPSS-R and IPSS-M, and a high risk of progression to leukemia independently of IPSS-M, which has not been reported in previous literature, as far as we know. Further, lower transthyretin is a simple and economic indicator for the prognostic measure of MDS, a useful supplement to the IPSS-R and IPSS-M, especially for MDS patients without karyotype or mutation detections. Our study had some limitations. Firstly, some cytokines and nutritional factors were not evaluated in this study. Secondly, our study lacked mutation data on all patients because gene mutation analysis of MDS patients in the early phase was not performed. Another limitation was that the analyzed data in this study originated from a single center.

In summary, decreased serum transthyretin could be an independent adverse prognostic factor in MDS patients. The transthyretin level, a simple and economic indicator for the prognostic measure of MDS, could provide a useful supplement to IPSS-R and IPSS-M, especially for MDS patients without karyotype or mutation detections. Clinically, timely nutritional intervention might improve the status of the body and prolong survival for MDS patients with low transthyretin levels in serum.

## Data availability statement

The original contributions presented in the study are included in the article, further inquiries can be directed to the corresponding authors.

## Ethics statement

The studies involving human participants were reviewed and approved by Human Ethics Committee of Ningbo First Hospital. The patients/participants provided their written informed consent to participate in this study.

## Author contributions

YC and TN were involved in the conception and design. TC, YW, and DZ was performed data acquisition. CS, YW, ZZ, NW, and LS was performed analysis and interpretation of the data. The drafting of the manuscript was revised critically for intellectual content by QM, XC, and GO. The remaining authors contributed to refining the ideas, carrying out additional analyses and finalizing this paper. All authors agree to be accountable for all aspects of the work.

## Funding

This research was supported by the Natural Science Foundation of Zhejiang Province (LY20H080001), the Medical and Health Science and Technology Projects of Zhejiang Province (2021KY283, 2021KY997, 2023KY263, and 2023KY1050), Natural Science Foundation of Ningbo Municipality (202003 N4228).

## Conflict of interest

The authors declare that the research was conducted in the absence of any commercial or financial relationships that could be construed as a potential conflict of interest.

## Publisher’s note

All claims expressed in this article are solely those of the authors and do not necessarily represent those of their affiliated organizations, or those of the publisher, the editors and the reviewers. Any product that may be evaluated in this article, or claim that may be made by its manufacturer, is not guaranteed or endorsed by the publisher.
